# *Miro1* depletion disrupts spatial distribution of mitochondria and leads to oocyte maturation defects

**DOI:** 10.3389/fcell.2022.986454

**Published:** 2022-10-17

**Authors:** In-Won Lee, Deepak Adhikari, John Carroll

**Affiliations:** Development and Stem Cell Program and Department of Anatomy and Developmental Biology, Monash Biomedicine Discovery Institute, Monash University, Melbourne, VIC, Australia

**Keywords:** mitochondrial transport, Miro1, oocyte, embryo development, mitochondrial adaptor protein

## Abstract

Mitochondria are dynamic organelles that undergo regulated microtubule- and actin-mediated trafficking to meet local energy and metabolic needs. Mitochondrial trafficking may be particularly critical in large cells such as eggs and early embryos where spindle formation and polar body extrusion occur in specific regions of the cytoplasm. To investigate the role of mitochondrial distribution in oocytes we have targeted the mitochondrial membrane protein, MIRO1, which couples mitochondria to the motor protein-TRAK complex. Oocyte-specific deletion of MIRO1 leads to the formation of large aggregates of mitochondria in perinuclear and cortical compartments. Mitochondria remain capable of long-range trafficking during maturation, indicating redundancy in the mechanisms coupling mitochondria to motor proteins. Polar body extrusion in the absence of MIRO1 was reduced by approximately 20%. In MIRO1-deleted zygotes, mitochondria showed increased accumulation around the pronuclei but this did not affect mitochondrial distribution to daughter blastomeres. *In vitro* development of parthenogenetic embryos was also reduced, although no differences were found in the fertility of oocyte-specific Miro1 KO mice. These findings demonstrate MIRO1 acts as a mitochondrial adaptor, setting mitochondrial distribution in oocytes and early embryos, and disrupting this process compromises *in vitro* oocyte maturation and embryo development.

## Introduction

Mitochondria play multiple roles in cells including the provision of energy in the form of ATP, the production of metabolites, and roles in controlling major cellular events including Ca^2+^ signaling and apoptosis. In recent years, the subcellular distribution of mitochondria has been shown to influence diverse cell functions including synaptic transmission, cell motility and ensuring inheritance of mtDNA in daughter cells (Klecker and Westermann, 2020; Moore et al., 2021). The distribution of mitochondria involves motor protein-driven trafficking on microtubules and actin filaments (Sheng and Cai, 2012). Long-range mitochondrial movement is typically attributed to microtubule-based dynein- or kinesin-driven trafficking, while actin-mediated movement occurs over shorter distances *via* myosin family motors and may be important for mitochondrial anchoring in specific subcellular domains (Ligon and Steward, 2000; Moore and Holzbaur, 2018; Cardanho-Ramos et al., 2020).

How mitochondria are coupled to motor proteins is becoming increasingly understood. The prevailing model involves mitochondrial outer membrane Rho GTPase, MIRO1/RHOT1, binding to TRAK/Milton which couples mitochondria to the motor protein (Gorska-Andrzejak et al., 2003; Fransson et al., 2006; Glater et al., 2006; Saotome et al., 2008; Brickley and Stephenson, 2011; Birsa et al., 2013; van Spronsen et al., 2013; Lopez-Domenech et al., 2018; Eberhardt et al., 2020). These adaptors provide another level of regulation for controlling the spatiotemporal distribution of mitochondria in response to different cell states and signaling pathways. At metaphase, CDK1 activity leads to the uncoupling of motor proteins and TRAK thereby freeing mitochondria for actin-mediated mixing and redistribution prior to cell division (Moore et al., 2021). In response to Ca^2+^ signaling at active synapses, MIRO acts as a Ca^2+^ sensor inhibiting kinesin-mediated trafficking (Macaskill et al., 2009) leading to the accumulation of mitochondria at active synapses where they play critical roles in calcium buffering and ATP provision (Saotome et al., 2008; Nemani et al., 2018; Eberhardt et al., 2020). MIRO can also regulate the main mitochondrial-actin motor, MYO19, ensuring stability to actin-mediated mitochondrial distribution (Lopez-Domenech et al., 2018; Oeding et al., 2018), while MIRO itself is subject to ubiquitination by the PINK/PARKIN pathway, effectively uncoupling damaged mitochondria from the cytoskeleton (Wang et al., 2011; Shlevkov et al., 2016; Safiulina et al., 2019). The wide-ranging modes of regulating mitochondrial traffic provide a sophisticated approach to ensure that mitochondria are distributed in a manner to best meet the needs of the cell.

This spatial organization is particularly relevant in large cells such as oocytes, in which ATP-consuming activities, including building the meiotic spindles and extruding polar bodies, take place in small subcellular compartments (Van Blerkom and Runner, 1984; Yu et al., 2010; Dalton and Carroll, 2013; Dalton et al., 2014). Oocyte mitochondria are essential because glycolysis is inhibited and mitochondrial oxidative phosphorylation is the sole source of oocyte-derived ATP (Biggers et al., 1967; Barbehenn et al., 1974; Saito et al., 1994; Li et al., 2020). This is consistent with mitochondria being highly mobile in mouse oocytes where they aggregate around the developing spindle and then undergo a redistribution through the cytoplasm in mature oocytes. Changes in mitochondrial distribution are accompanied by changes in ATP levels during maturation suggesting that accumulation of mitochondria may lead to increased ATP for events such as spindle formation and polar body extrusion (Yu et al., 2010). Dynein and Kinesin-based trafficking mechanisms are responsible for distributing mitochondria in oocytes (Dalton and Carroll, 2013; Adhikari et al., 2022), but the role of these motors in many cellular events, including spindle formation and function, preclude them as targets for experimentally manipulating mitochondrial distribution. We have therefore targeted *Miro1* as a strategy to alter the mitochondrial distribution and ask if it is critical for events of oocyte maturation and early development.

Mammals express two MIRO orthologs, MIRO1 and MIRO2 that show 60% sequence similarity (Fransson et al., 2003; Fransson et al., 2006). Recent studies have shown that *Miro1* knockout animals die perinatally, whereas *Miro2* knockout animals can develop normally and survive until adulthood, suggesting essential roles of MIRO1 for development (Nguyen et al., 2014; Lopez-Domenech et al., 2016; Lopez-Domenech et al., 2018). We have generated oocyte-specific *Miro1* knockout mice in an effort to sever mitochondria from motor proteins to investigate the role of mitochondrial distribution on egg and embryo development. Our results show that deletion of *Miro1* in mouse oocytes alters the mitochondrial structure and alters long-distance mitochondrial transport during maturation. Functionally, lack of *Miro1* impairs oocyte maturation and embryo development *in vitro* but appears to be dispensable for successful preimplantation embryo development *in vivo*.

## Materials and methods

### Animals and genotyping

All animal experiments were approved by Monash University Animal Ethics Committee and were performed in accordance with Australian National Health and Medical Research Council Guidelines on Ethics in Animal Experimentation. Floxed *Miro1* (*B6(Cg)-Rhot1* ^*tm2.1Jmsu*^ */J*, stock number 031126) transgenic line in C57BL/6 background was obtained from the Jackson laboratory (Nguyen et al., 2014). To produce mice with oocyte-specific *Miro1* deletion, *Miro1* ^*F*^ mice were crossbred with transgenic mice carrying a *ZP3* promoter-mediated Cre recombinase (*Miro1* ^*F*^ *; ZP3-Cre* or *Miro1* ^*cKO*^). CBBF1 males were used in breeding experiments. Animals were housed under controlled environmental conditions where water and food were provided *ad libitum*.

Tail tissue was collected for genotyping. Genotypes were confirmed by a real-time PCR-based assay outsourced to Transnetyx.

### Oocyte collection

Germinal vesicle (GV)-stage oocytes were collected from the ovaries of 4 to 5-week-old female mice previously primed (44–48 h) with an intraperitoneal injection of pregnant mare’s serum gonadotropin (PMSG, 10 IU; ProSpec). Isolated ovaries were placed in a pre-warmed M2 medium (Sigma-Aldrich) at 37°C. The M2 medium used for oocyte collection contained 200 µM of 3-isobutyl-1-methylxanthine (M2 + IBMX; Sigma-Aldrich) to inhibit spontaneous GV breakdown during handling. Ovarian follicles were punctured with a 27-gauge syringe needle to release cumulus-oocyte complexes (COCs). A narrow bore glass pipette was used with repetitive pipetting to remove cumulus cells surrounding fully grown GV oocytes. For *in vitro* maturation, GV-stage oocytes were washed in M16 medium (Sigma-Aldrich) before being transferred to drops of the same medium covered with mineral oil in an incubator at 37°C (5% CO_2_ in air).

Super-ovulated MII-stage oocytes were collected from the oviducts after human chorionic gonadotropin (hCG, 10 IU; Sigma-Aldrich) injection at 44–48 h post PMSG stimulation. Oocytes were incubated in M2 medium containing 300 μg/ml hyaluronidase (Sigma-Aldrich) for 1 min to remove cumulus cells and cultured in M2 medium until further experiments.

### Parthenogenetic activation and embryo culture

Collected MII-stage eggs were cultured in calcium-free CZB medium (81.62 mM NaCl, 4.83 mM KCl, 1.18 mM MgSO_4_, 0.11 mM EDTA, 5.6 mM glucose, 37 mM sodium lactate, 1.2 mM KH_2_PO_4_, 25.1 mM NaHCO_3_, 0.27 mM pyruvate, 1 mM l-glutamine, 5 mg/ml BSA, pH 7.4) with 10 µM Cytochalasin D (Sigma-Aldrich) and 5 mM SrCl_2_ (Sigma-Aldrich) for 3 h in the incubator at 37°C (5% CO_2_ in air). Eggs were further incubated in M2 medium supplemented with 10 µM Cytochalasin D for 2 h on the heat block set at 37°C. For *in vitro* culture, parthenogenetically activated eggs were washed in SAGE-1 Step medium (CooperSurgical) before being transferred to drops of the same medium covered with mineral oil in a humidified atmosphere of 5% CO_2_ in air at 37°C.

### Analysis of RNA sequencing data

Published unprocessed RNA-seq reads of mouse oocyte and early embryo transcriptome data were obtained from NCBI Sequence Read Archive (SRA, dataset accession number: SRP105271) (Wang et al., 2018). This dataset was sequenced on an Illumina HiSeq 2500 (paired-end 125-bp or 150-bp). Gene expression was quantified using Kallisto (Bray et al., 2016) and Sleuth (Pimentel et al., 2017). Read quantification was performed with Kallisto, a pseudo-alignment-based method to quantify RNA abundance at the transcript level in transcripts per million (TPM) counts. Kallisto quant was utilized with the number of bootstraps set to 100 using ENSEMBL cDNA transcripts (Mus_musculus.GRCn38). Differential gene expression was performed using Sleuth in R to leverage the bootstrap estimates of Kallisto and to output gene-level normalized TPM.

### Immunofluorescence and imaging

Oocytes were fixed in 4% paraformaldehyde (PFA) for 30 min at room temperature and permeabilized in 0.5% Triton X-100 for 30 min. After 1 h of blocking in 3% BSA in PBS, oocytes were stained with the primary antibodies overnight at 4°C; anti-rabbit RHOT1 (NBP1-59021, Novus), anti-mouse MTCO1 (ab14705, Abcam), and anti-rabbit CDX2 (ab76541, Abcam). After washing three times in PBS containing 0.01% Triton X-100 and 0.1% Tween 20, they were labeled with the secondary antibodies, Alexa Fluor 488, 555, 568, or 647 for 2 h at room temperature. Meiotic spindles were stained using an Alexa fluor conjugated anti-ɑ-Tubulin antibody (#322588, Invitrogen) at a concentration of 1/200 in 1% BSA. DNA was labeled by Hoechst 33342 (10 μg/ml; Sigma-Aldrich) for 10 min at room temperature. After washing three times, oocytes were placed in PBS-PVA drops on the glass-bottomed fluorodish (World precision instruments) for imaging on a Leica SP8 confocal or Zeiss ZSM980 airy scan microscope.

### Mitochondrial staining for live cell imaging

To label mitochondria in live cells, oocytes were cultured in M2 medium supplemented with 25 nM Tetramethylrhodamine, Methyl Ester, Perchlorate (TMRM, Invitrogen) at 37°C for 45 min. After washing three times in fresh M2 medium, oocytes were transferred to drops of M2 medium containing 5 nM TMRM on a round coverslip inserted into a 35 mm metal chamber. TMRM signals were captured using an inverted Leica SP8 confocal microscope equipped with a temperature-controlled incubation box. Single planes of midsections or full z-stacks of whole oocytes were imaged at 1 or 1.5 µm intervals.

### Analysis of mitochondrial clusters

Z-stack images of TMRM-stained oocytes were pre-processed using Fiji software. Briefly, a gaussian filter was set to 1 to reduce noise and enhance features of mitochondrial clusters. To measure the volume and count the number of mitochondrial clusters, 3D surface rendering of mitochondrial clusters was created by Imaris software (Bitplane) with certain processing settings. For area fraction analysis, the mitochondrial signal was measured using a basic measurement function in Fiji after adjusting the threshold.

### Transmission electron microscopy

GV-stage oocytes were fixed with 2.5% glutaraldehyde in 0.1 M sodium cacodylate (pH 7.4) overnight in the cold room and were washed three times with a cacodylate buffer. Samples were post-fixed with 2% osmium tetroxide and 1.5% potassium ferricyanide for 2 h at room temperature and biowaved in 1% Thiocarbohydrazide, 2% osmium tetroxide, 2% uranyl acetate, and lead aspartate, respectively, three times for 2 min at 100 W. To dehydrate, oocyte samples were biowaved with ethanol 50%, 70%, 9%, 100% each for 60 s at 150 W. Then, samples were infiltrated with propylene oxide:hard epon (1:1), propylene oxide:hard epon (1:3), and 100% hard epon each for 3 min at 250 W in the biowave and polymerized in Beem capsules in the oven at 60° for 48 h. Resin blocks containing samples were trimmed with a Diatome diamond knife and processed on the Jeol 1400 plus transmission electron microscope.

### Analysis of mitochondrial size

The area (size, µm^2^) of individual mitochondria on single-plane images was measured using Fiji software. Measured values obtained from control oocytes were applied by the Shapiro-Wilk test for log-normality and normally distributed (Average = 0.13 µm^2^, Standard deviation = 0.08). Then, each mitochondrion was binned into a Large (>0.13 + 0.08 µm^2^), Intermediate, or Small (<0.13–0.08 µm^2^) group, and the number of classified mitochondria was counted in control or Miro1 KO oocytes. Statistical analysis was performed by one-way ANOVA followed by Wilcoxon signed-rank test.

### *In vitro* transcription and microinjection

To generate capped mRNA, the linearized plasmid template was transcribed using T3 mMessage mMachine Transcription kit (Invitrogen), polyadenylated by Poly(A) tailing kit (Invitrogen) and purified with RNeasy Mini Cleanup kit (Qiagen): GFP-Cyclin B1 (200 ng/μl).

GV-stage oocytes were microinjected with the indicated cRNA using a micropipette and an MMN-1 coarse micromanipulator (Narishige) mounted on an Axiovert 15 inverted microscope (Zeiss). Oocytes were placed in a drop of M2 + IBMX covered with mineral oil on the lid of a 35 mm Petri dish. A holding pipette (Gytech) was used to position the oocytes for microinjection and a micropipette (Harvard Apparatus) was inserted into the oocyte cytoplasm. An intracellular electrometer was used to allow a brief pulse of negative capacitance to be applied across the plasma membrane to assist the pipette tip to breach the membrane. Then, cRNA was pressure-injected into the cytoplasm of fully grown GV-stage oocytes using a fixed pressure pulse through a PV820 pneumatic picopump (World precision instruments). Microinjection volume was estimated at 5% by cytoplasmic displacement. Following microinjection, oocytes were placed in a fresh drop of M2 medium covered by mineral oil at room temperature for 5 min and moved to 37 °C heat blocks for several min to allow the oocytes to recover.

### Statistical analysis

Statistical analysis of experiments with two groups was carried out using an unpaired *t*-test or paired *t*-test accordingly. For the comparisons of the polar body extrusion and blastocyst formation, Chi-square (and Fisher’s exact) test was used. Data are expressed as the mean ± SD. On the violin and whisker plots, the central line indicates median and the top and bottom of the line indicate 25% and 75%. * denotes a *p*-value of <0.05, ** denotes a *p*-value of <0.01, *** denotes a *p*-value of <0.001, and *** denotes a *p*-value of <0.0001.

## Results

### *Miro1* is highly expressed and localized to mitochondria in mouse eggs

**FIGURE 1:**
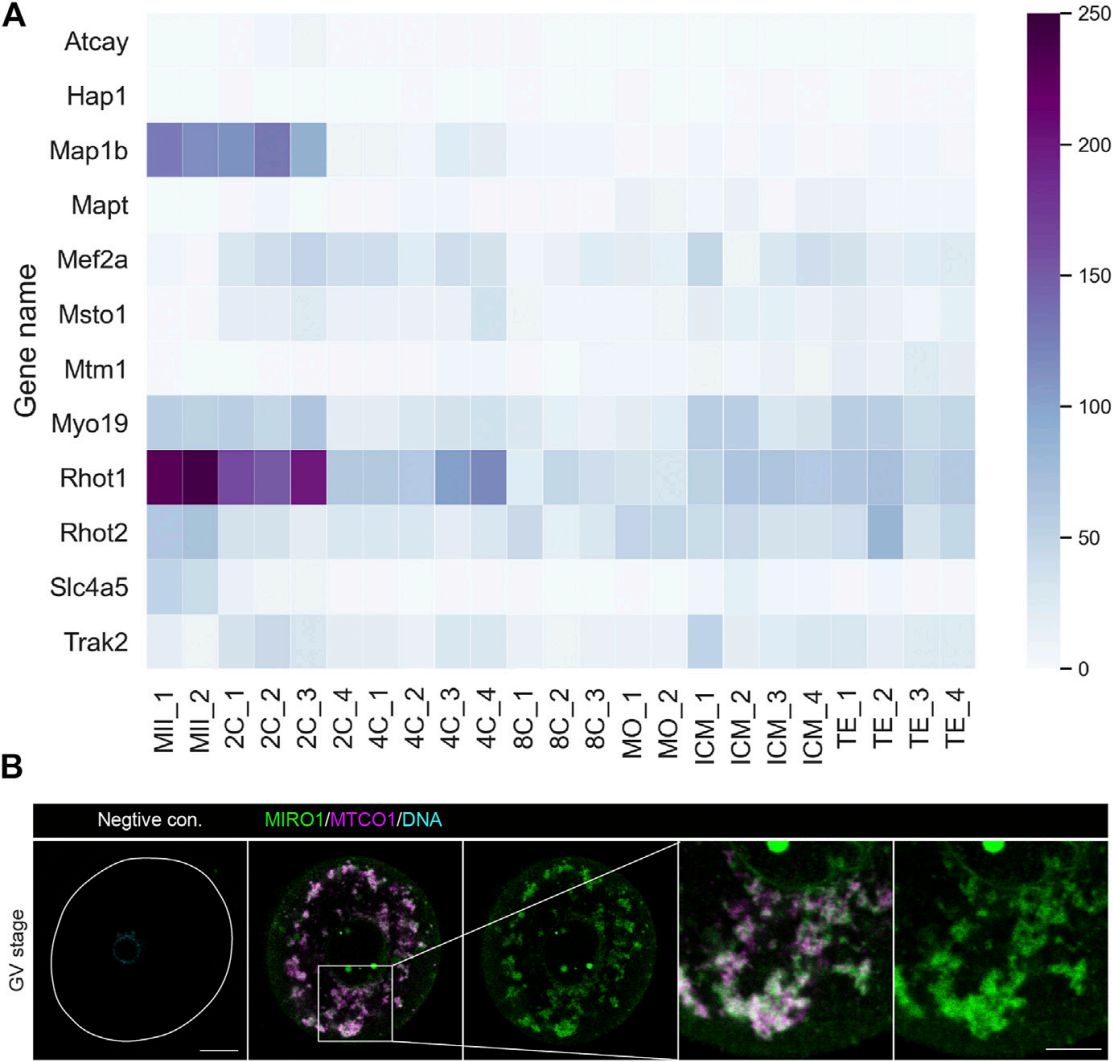
MIRO1 protein localization in mitochondria of mouse oocytes**. (A)** A heatmap of the expression levels of transcripts related to mitochondrial transport genes. Results are in transcripts per million (TPM) with blue range colors. **(B)** Representative images of the localization of MIRO1 in a fully grown GV-stage oocyte. Green: MIRO1, Magenta: MTCO1, Cyan: Hoechst 33342. Scale bar: 15 µm in whole oocytes and 10 µm in cropped images.

Before conducting experiments in oocytes, we analyzed the mRNA levels of *Miro* and related genes from previously published transcriptomics data in mouse eggs (Wang et al., 2018). A heat map shows the transcripts classified under Gene Ontology annotation GO:0048311 (mitochondrion distribution), GO:0047497 (mitochondrion transport along microtubule) and GO:0034642 (mitochondrion migration along actin filament) were expressed in both mature oocytes and early stage embryos (Figure 1A). *Rhot1* (ortholog *Miro1* in mammals) in particular and *Rhot2* (orthologue *Miro2* in mammals) transcripts were expressed at relatively high levels compared to other mitochondrial transport machinery-related genes. Next, we performed immunostaining of MIRO1 together with a mitochondrial marker MTCO1 (Figure 1B) and found MIRO1 and MTCO1 proteins to be tightly co-localized, indicating potential roles of MIRO1 in oocyte mitochondrial function.

As MIRO1 is expressed at relatively high levels and localized to oocyte mitochondria, we hypothesized that deletion of *Miro1* would impair mitochondrial distribution in mouse oocytes and allow us to ask if this impacts oocyte and early embryo development. Because *Miro1* knockout animals die perinatally due to a breathing dysfunction at birth (Turgeon and Meloche, 2009; Nguyen et al., 2014; Lopez-Domenech et al., 2016), we generated an oocyte-specific *Miro1* conditional knockout line, by crossing *Miro1* ^*F*^ and oocyte-specific *ZP3-Cre* mice (Supplementary Figure S1A). Exon 2 (encoding part of the GTPase I domain) of the mouse *Miro1* gene is flanked by loxP sites in *Miro1* ^*F*^ mice and *Miro1* inactivation was achieved by crossing these mice with a mouse line expressing Cre recombinase from the oocyte-specific ZP3 promoter. For simplicity, eggs from mice with a genotype *Miro1* ^*F*^ are hereafter referred to as controls, and eggs from *Miro1* ^*F*^ *; ZP3-Cre* mice are referred to as Miro1 KO.

To confirm the successful deletion of *Miro1* in *Miro1* ^*F*^ *; ZP3-Cre* mouse oocytes, PCR analysis of DNA extracted from pooled GV oocytes was performed with primers P1, P2 and P3 as previously designed by the original investigator (Nguyen et al., 2014) (Supplementary Figures S1A,B). Endogenous MIRO1 protein levels were quantified in controls and Miro1 KO oocytes by immunofluorescence (Supplementary Figures S1C,D). The MIRO1 fluorescence was clearly observed in control mitochondria but it was almost completely absent in mitochondria of Miro1 KO oocytes, demonstrating an effective deletion of MIRO1 in Miro1 KO oocytes.

### Changes in mitochondrial distribution and structure in *Miro1* null oocytes

**FIGURE 2:**
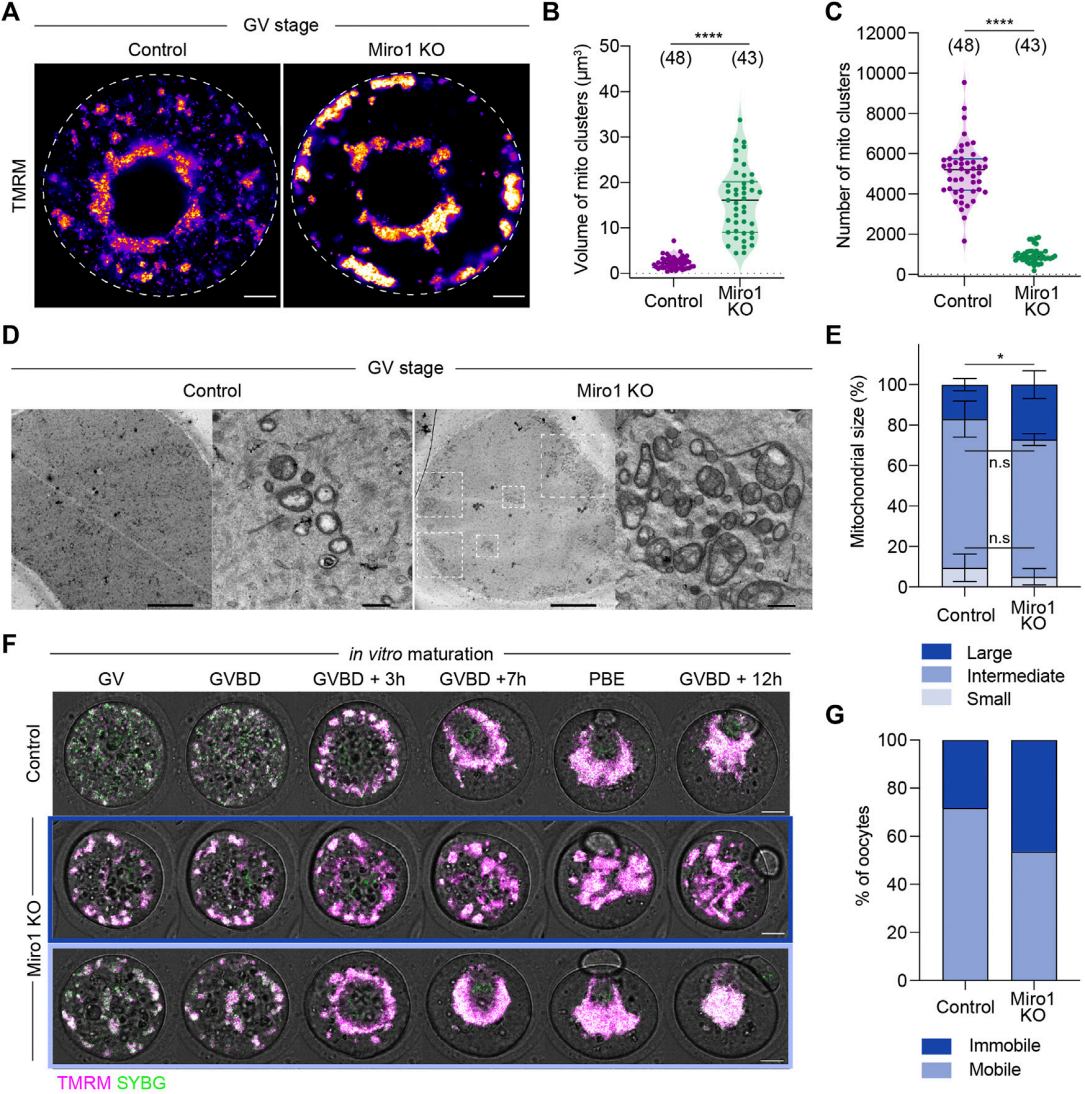
Deletion of *Miro1* alters mitochondrial localization and mitochondrial ultrastructure. **(A)** Representative images of mitochondrial localization in GV-stage control and Miro1 KO. Fire: TMRM. Scale bar: 10 µm. **(B,C)** Comparison of volume and number of mitochondrial clusters in control and Miro1 KO. Unpaired *t*-test ****p < 0.0001. Data are collated from 3 independent experimental replicates **(D)** TEM images of whole oocytes and high magnified mitochondria in control and Miro1 KO. Dashed square: Mitochondrial aggregates. Scale bar of whole oocyte image: 10 μm, magnified: 500 nm. **(E)** Semi-quantification analysis of individual mitochondria observed in TEM images. Control (*n* = 455) or Miro1 KO (*n* = 608). One-way ANOVA followed by Wilcoxon signed-rank test. *p < 0.05. Data are collated from 3-4 independent experimental replicates and presented as mean ± SD **(F)** Time-lapse images of maturing control and Miro1 KO. Magenta: TMRM, Green: Sybr Green. Scale bar: 15 µm. **(G)** Percentage of control (*n* = 39) or Miro1 KO (*n* = 41) having mobile or immobile mitochondria. Chi-square test, n.s., not significant, *p* = 0.09. Data are collated from 3 independent experimental replicates.

Relatively homogeneously distributed mitochondria in the cytoplasm of control GV oocytes indicates a balanced motor protein activity (Dalton and Carroll, 2013). We hypothesized that mitochondrial organization would be disrupted in Miro1 KO oocytes due to the uncoupling of mitochondria from cytoskeletal tracks. We investigated the effects of deleting *Miro1* on mitochondrial distribution in live TMRM-labeled oocytes. Miro1 KO oocytes showed a marked difference in mitochondrial distribution and organization as compared to controls. First, control oocytes showed a typical mitochondrial distribution through the cytoplasm with some concentration in the perinuclear region (Figure 2A). In contrast, almost all mitochondria in Miro1 KO oocytes became localized to the perinuclear region and to the oocyte cortex, resulting in the clearance of mitochondria from much of the oocyte cytoplasm (Figure 2A). Second, in Miro1 KO oocytes, mitochondria became highly aggregated into a small number of large clusters, while in controls mitochondria remained dispersed in a large number of small clusters (Figures 2B,C). Third, transmission electron microscopy (TEM) studies revealed that Miro1 KO oocytes contained a higher number of larger mitochondria than control oocytes (Figures 2D,E). Additionally, mitochondria in Miro1 KO oocytes contained more complex cristae structures compared to the typical small vacuolated mitochondria of controls (Figure 2D). These results show that deletion of *Miro1* significantly alters the distribution and morphology of mitochondria in GV-stage oocytes.

As oocytes show dramatic changes in mitochondrial redistribution during maturation (Van Blerkom and Runner, 1984; Dalton and Carroll, 2013), we examined if this was disrupted in Miro1 KO oocytes. To compare mitochondrial redistribution, we performed time-lapse imaging of TMRM-labeled control and Miro1 KO oocytes during maturation (Figure 2F). In controls, mitochondria were initially dispersed through the cytoplasm before aggregating around the developing spindle in pro-metaphase I (Figure 2F). Then, mitochondria migrated to the cortex with the spindle and redistributed through the cytoplasm after polar body extrusion (PBE). In contrast, there were two distinct patterns of mitochondrial movements observed in Miro1 KO oocytes (Figures 2F,G). First, 54% of Miro1 KO oocytes showed the typical aggregation of mitochondria around the spindle. Because mitochondria in Miro1 KO oocytes were already aggregated as cytoplasmic clusters, the spindle association was achieved by the movement of the entire cluster. This resulted in highly effective clearance of mitochondria from the cytoplasm to the spindle region. Second, in the rest (46%) of Miro1 KO oocytes, the clusters of mitochondria remained in their original location and only a limited spindle aggregation was apparent (Figures 2F,G). These results show that deletion of *Miro1* significantly alters mitochondrial distribution and trafficking associated with oocyte maturation.

### *Miro1* deletion leads to changes in mitochondrial function

To examine if *Miro1* deletion and the resultant changes in mitochondrial organization impact mitochondrial function, we assessed several functional parameters including NADH, FAD^2+^, reactive oxygen species (ROS), ATP levels, and mitochondrial membrane potential (MMP). Mitochondrial NADH and FADH_2_ are generated by TCA cycle and then oxidized by Complex I and Complex II of the electron transport chain (ETC), respectively. We found the level of FAD^2+^ was markedly increased in fully grown Miro1 KO oocytes, indicating an increase in Complex II activity or reduced TCA cycle activity. In contrast, NADH levels were unchanged (Supplementary Figures S2A,B) as were the levels of ROS and ATP (Supplementary Figures S2C,D). We also measured MMP using a ratiometric approach with an MMP-insensitive mitochondrial marker (Mitotracker Green) and an MMP-sensitive TMRM (Supplementary Figures S2E,F) (Al-Zubaidi et al., 2019). The level of MMP in Miro1 KO oocytes was significantly lower compared to controls despite increased mitochondrial aggregation in Miro KO oocytes leading to strong mitotracker green and TMRM intensities. Taken together, mitochondrial ETC and TCA cycle activities may be altered as a result of *Miro1* deletion, while functional parameters such as ROS and ATP remain unaltered.

### *Miro1* deletion impairs *in vitro* oocyte maturation and results in ovulated oocytes with altered mitochondrial organization

**FIGURE 3:**
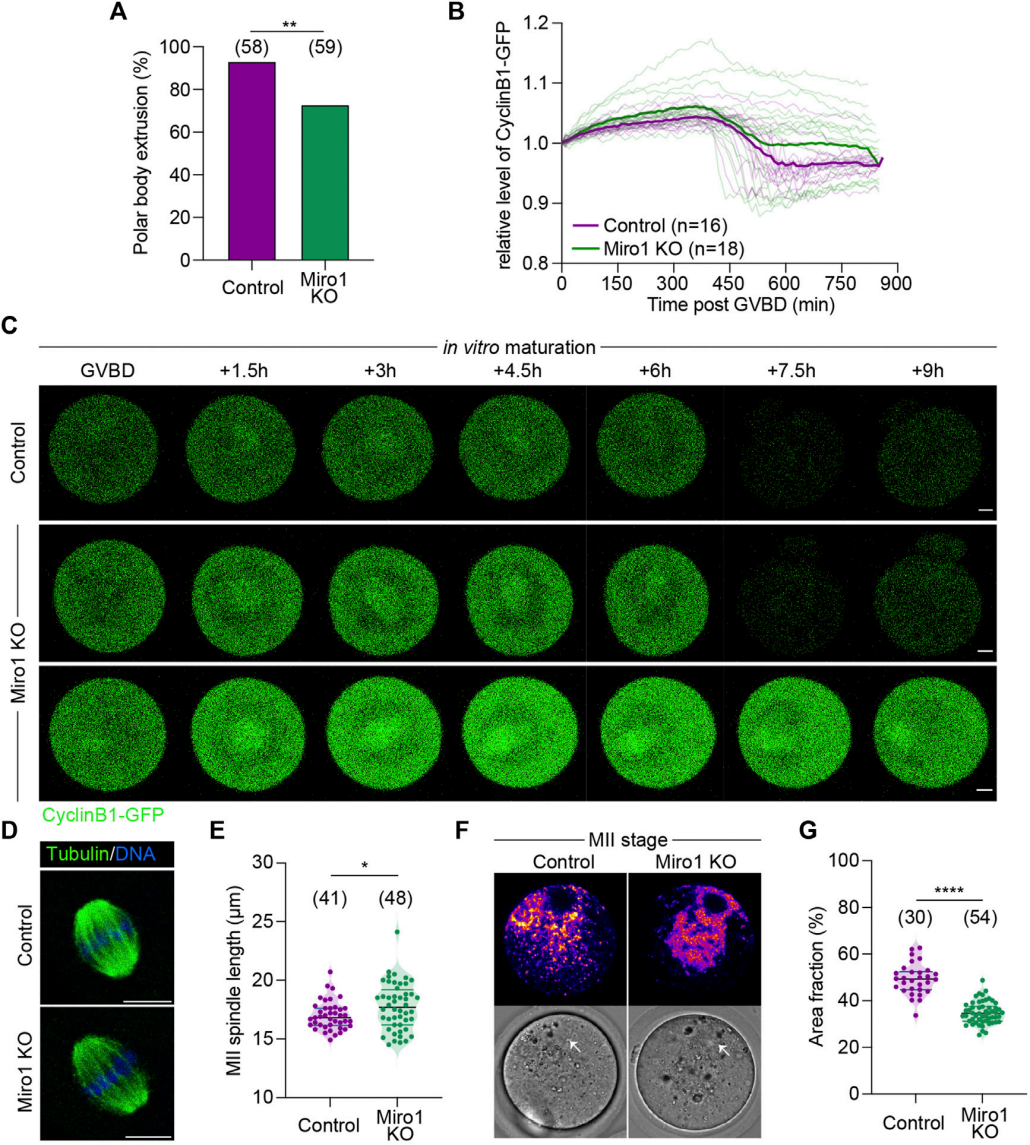
Effects of Miro1 deletion on mouse oocyte maturation. **(A)** Percentage of the first polar body extrusion in control and Miro1 KO. Chi-square **p < 0.01. **(B)** Normalized fluorescence intensity of Cyclin B1-GFP during oocyte maturation in control (*n* = 16) and Miro1 KO (*n* = 18). Paired *t*-test, ****p < 0.0001 **(C)** Time-lapse image of maturing control and Miro1 KO. Green: Cyclin B1-GFP. Scale bar: 10 µm. **(D)** Morphology of bipolar spindles observed in ovulated controls and Miro1 KO. Green: a-Tubulin and Blue: DNA. Scale bar: 10 µm **(E)** Length of the second meiotic spindle. Unpaired *t*-test *p < 0.05. Data are collated from 3 independent experimental replicates. **(F)** Mitochondrial distribution in ovulated controls and Miro1 KO. Fire: TMRM. White arrow: spindle. Scale bar: 10 µm **(G)** Percentage of area fraction measured at ROIs (white dashed line) in control and Miro1 KO. Unpaired *t*-test ****p < 0.0001. Data are collated from 3 independent experimental replicates.

Given the effect of *Miro1* deletion on mitochondrial structure and function in GV oocytes, we examined the ability of Miro1 KO oocytes to undergo maturation *in vitro*. The rate of oocyte maturation as evidenced by first polar body extrusion (PBE) was decreased by approximately 20% in Miro1 KO compared to control oocytes (72.9% in Miro1 KO vs. 93.1% in control; Figure 3A). To examine if this difference was caused by failure to make the transition from metaphase I to anaphase/telophase I, the degradation of Cyclin B1-GFP was monitored during maturation (Figures 3B,C). In a proportion of Miro1 KOs (63.3%), Cyclin B1 was not degraded resulting in a higher average level of Cyclin B1-GFP (Figure 3C), consistent with the arrest at metaphase I and lower PBE rates.

Given this modest but significant impact on oocyte maturation *in vitro*, we examined the spindle and mitochondrial organization in ovulated Miro1 KO oocytes. Analysis of immunofluorescence of MII spindles in ovulated oocytes revealed that the overall barrel-shaped spindle organization and chromosome alignment was not affected by the lack of *Miro1*, although spindle length was slightly increased in Miro1 KO oocytes (Figures 3D,E). Analysis of mitochondrial distribution in ovulated Miro1 KO oocytes showed that mitochondria were mostly aggregated in the oocyte central cytoplasm just below the cortically localized MII spindle, with a small focal cluster of mitochondria reaching up to encapsulate the spindle (Figure 3F). Controls showed the typical MII distribution where mitochondria are dispersed across the spindle hemisphere including across the cortical domain (Figure 3F). Accordingly, the central cytoplasmic aggregation in Miro1 KO oocytes resulted in the fractional area occupied by mitochondria in an equatorial optical section being decreased compared to controls (Figure 3G). Overall, the lack of MIRO1 in oocytes causes altered mitochondrial structure and localization and leads to detectable changes in oocyte maturation *in vitro*.

### Deletion of *Miro1* alters mitochondrial distribution during preimplantation development but is dispensable for embryogenesis

**FIGURE 4:**
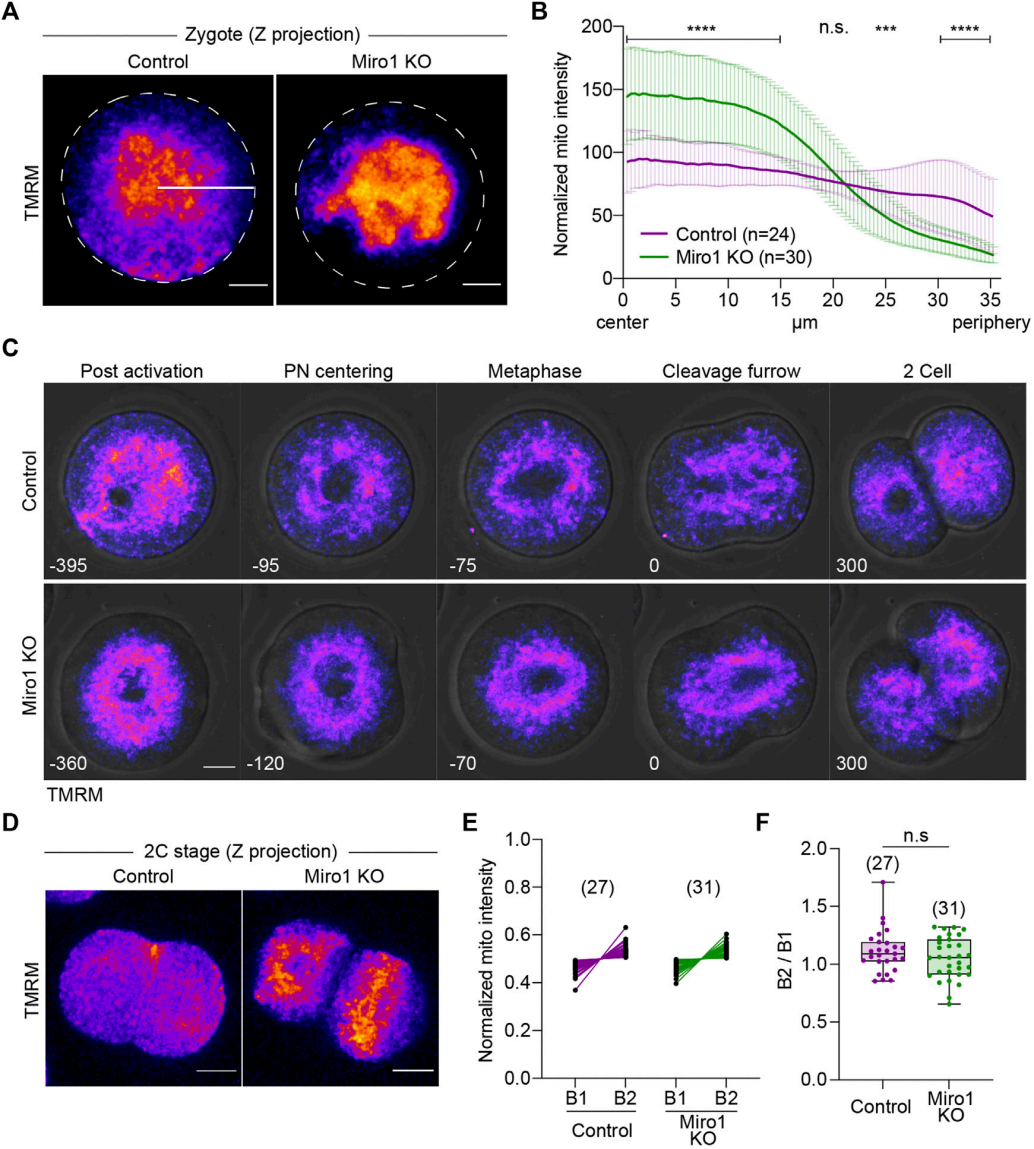
Loss of Miro1 results in severe mitochondrial disorganization in early parthenotes**. (A)** Representative images of 1-cell parthenotes were imaged after the activation. Fire: TMRM. Dashed line: Oocyte membrane. White line: ROI (35 µm long) for analysis. Scale bar: 15 µm **(B)** Normalized mitochondrial intensity from the center to the cell periphery (35 µm) in a circle (360°). Data presented as mean ± SD. Paired *t*-test, ****p < 0.0001, ***p < 0.001, n.s., not significant. Data are collated from 3 independent experimental replicates **(C)** Mitochondrial distribution during the first mitotic cell division in control and Miro1 KO parthenotes. Fire: TMRM. Time: min. Scale bar: 15 µm. **(D)** Representative images of mitochondrial distribution in 2-cell parthenotes. Fire: TMRM. Scale bar: 15 µm. **(E)** Quantification data of mitochondrial inheritance in each blastomere at 2-cells. Normalized mitochondrial intensity was measured in z-projection images. **(F)** Ratio of mitochondrial intensity in B1 and B2. Unpaired *t*-test, n.s., not significant. Data are collated from 3 independent experimental replicates.

Given the significant effects of *Miro1* deletion on mitochondrial organization and oocyte maturation, we next studied if MIRO1 plays a role during preimplantation development. Parthenogenetic activation was used to address this question because the development of diploid parthenogenetic embryos to the blastocyst stage *in vitro* is similar to fertilized embryos (Balakier and Tarkowski, 1976). Additionally, parthenogenesis avoids the paternal allele thereby allowing the study of preimplantation development of homozygous *Miro1* (−/−) embryos. First, we examined mitochondrial distribution in parthenogenetic 1-cell and 2-cell embryos. In control zygotes, mitochondria aggregated at the center but mitochondria were also present in the cortical cytoplasm (Figure 4A). In contrast, in Miro1 KO zygotes examined 5–6 h after activation, mitochondria were aggregated at the center of the zygote, tightly surrounding the pronuclei. This clustering effectively cleared the cortical cytoplasm of mitochondria (Figure 4A). To analyze the changes in mitochondrial distribution in control and Miro1 KO zygotes, the average mitochondrial intensity from the center to the periphery of the zygote was measured using a Radial Profile Angle (radius set to 35 µm and the line ROI profiled over 360°). In control zygotes, the intensity of mitochondrial fluorescence was relatively consistent across the ROI indicating a homogeneous distribution (Figures 4A,B). In contrast, in Miro KO zygotes the mitochondrial distribution was significantly higher at the center (referred to as radius = 0) and fell precipitously to cortical cytoplasm (Figure 4B). This pattern of distribution is markedly different from that seen in Miro1 KO GV oocytes, where nearly half of the oocytes showed a cortical accumulation of mitochondria (Figure 2A). These data show that MIRO1 contributes to mitochondrial localization in zygotes, indicating its potential role during the transition from meiosis to mitosis.

Based on the altered mitochondrial distribution in 1-cell stage Miro1 KO, we hypothesized that mitochondria would be unevenly inherited across the blastomeres during the first mitotic cell division. To test this, we monitored mitochondrial movement during the transition from 1-cell to 2-cell using time-lapse imaging (Figure 4C). In control zygotes, mitochondria began to gather and center around the female and male pronuclei, surrounded the first mitotic spindle, and finally segregated into daughter cells (Figure 4C, top panel). In Miro1 KO zygotes, however, mitochondria mostly clumped in the middle of the cell with no mitochondria near the cell periphery (Figure 4C, bottom panel). To quantify mitochondrial inheritance into each daughter cell, the intensity of TMRM signal in 2-cell stage control and Miro1 KO parthenotes was measured in maximum z-projection images. Surprisingly, contrary to the expectation, mitochondria were fairly evenly distributed in the blastomeres of 2-cell Miro1 KO (Figures 4D–F).

**FIGURE 5:**
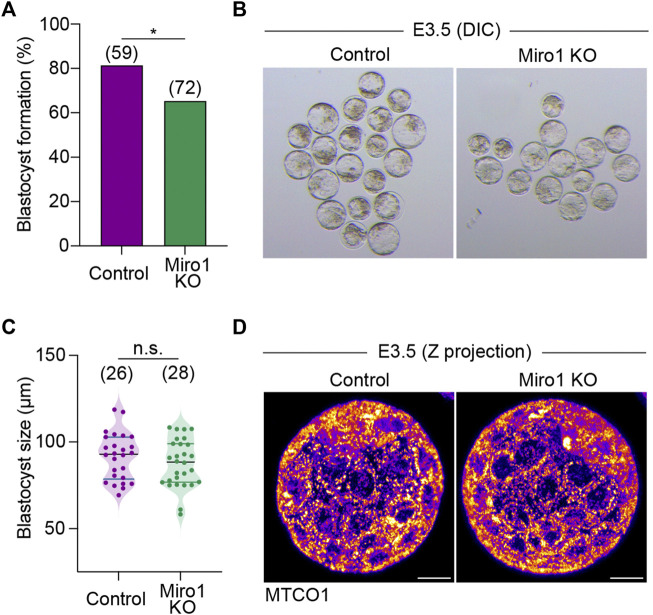
Miro1 KO parthenotes develop to blastocyst**. (A)** Percentage of blastocyst formation in control and Miro1 KO parthenotes. Chi-square *p < 0.05. Data are collated from 3 independent experimental replicates. **(B)** Representative DIC images of control and Miro1 KO parthenotes at E3.5. **(C)** Measurement of control and Miro1 KO blastocyst size. Unpaired *t*-test n.s., not significant. Data are collated from 3 independent experimental replicates. **(D)** Representative images (maximum z-stack projection) labeled with TMRM in control and Miro1 KO parthenotes at E3.5 (Representative of 26–28 parthenotes). Scale bar: 20 µm.

Next, we examined the roles of MIRO1 on development to the blastocyst stage. The majority of Miro1 KO parthenotes developed to blastocysts, albeit at a reduced rate compared to controls (65.3% in KO v 81.4% in control, Figures 5A,B). Control and Miro1 KO parthenote blastocysts were also comparable in size (Figure 5C) and had similar patterns of mitochondrial distribution (Figure 5D). Taken together, these data suggest that although the deletion of *Miro1* significantly alters mitochondrial distribution in oocyte and one-cell zygotes, MIRO1 function is less important in preimplantation development than in oocytes.

To study the fertility of *Miro1* ^*F*^ *;ZP3-Cre* mice, *Miro1* ^*F*^ *;ZP3-Cre* and *Miro1* ^*F*^ control females were mated with stud males. Both *Miro1* ^*F*^ and *Miro1* ^*F*^ *;Zp3-Cre* female mice regularly produced offspring with comparable litter sizes (Supplementary Figure S3). Additionally, the offspring from *Miro1* ^cKO^ were found to be viable until adulthood (not shown). These data suggest that MIRO1 in the oocyte is dispensable for oocyte growth and subsequent embryo development *in vivo*, however, *Miro1*-deficient oocytes are more sensitive to *in vitro* culture conditions and it leads to lower rates of oocyte maturation compared to control oocytes. Finally, to ask if MIRO2 may compensate for the loss of MIRO1, we microinjected *Miro2* siRNA into Miro1 KO oocytes and studied maturation of oocytes lacking both MIRO1 and MIRO2. We first confirmed that siMiro2 effectively degrades *Miro2* mRNA in both control and Miro1 KO oocytes (Supplementary Figure S4A). However, the first polar body extrusion rates of Miro KO oocytes injected with siMiro2 were not significantly different from Miro1 KO oocytes injected with control siRNA (Supplementary Figure S4B). These results suggest that MIRO2 is unlikely to compensate for the loss of MIRO1 in Miro1 KO oocytes.

## Discussion

Mitochondrial function is essential for oocyte and early embryo development. However, if the subcellular distribution of mitochondria plays a role in meeting local energy needs has not been addressed. Here, we have used oocyte-specific deletion of *Miro1* to examine its effect on mitochondrial trafficking and the developmental capacity of mouse eggs and early embryos. Our results show that deletion of *Miro1* alters the mitochondrial distribution normally associated with oocyte maturation and early embryo development, and that Miro1 KO oocytes and parthenogenetic embryos are more sensitive to *in vitro* culture environment, while *in vivo* development appears unaffected.

Consistent with the established role of MIRO1, its absence from oocytes altered the normal mitochondrial distribution. In immature Miro1 KO oocytes, one of the main features was that mitochondria clustered into a small number of large aggregates, rather than staying relatively dispersed through the cytoplasm. It is unclear how these aggregates come about. One possibility is that as fission takes place during oocyte growth, the daughter mitochondria remain in a cluster rather than being subject to short- and long-range trafficking on actin or microtubules respectively. Because these aggregates are primarily located in the cortex or perinuclear regions of GV-stage oocytes, it follows that these regions would have to preferentially support mitochondrial fission.

An alternative explanation for this aggregation and pattern of distribution is that fission is randomly distributed in the cytoplasm and that microtubule-mediated trafficking in both directions is increased. This appears paradoxical to the expected effect of *Miro1* deletion but could come about if MIRO1 normally suppresses microtubule-mediated trafficking, perhaps by anchoring mitochondria to the extensive cytoplasmic actin network. In addition, even in *Miro1/2* double knockdown cells, mitochondria remain aligned with microtubules, suggesting MIRO is not necessary for microtubule-mediated trafficking (Babic et al., 2015). It is postulated that TRAK adaptor proteins bind to other mitochondrial outer membrane proteins such as Mitofusin 1 (MFN1) (Misko et al., 2010; Lee et al., 2018; Lopez-Domenech et al., 2018). In Miro1 KO oocytes, the ability of mitochondrial aggregates to accumulate to the spindle region of maturing oocytes and the perinuclear region of zygotes shows that microtubule-mediated trafficking is active, but further work is needed to understand if this trafficking is driven by or other mitochondrial membrane adaptor proteins such as MFN1.

The stage-dependent differences in mitochondrial distribution; between immature and mature oocytes and between GV-stage oocytes and zygotes indicate both the cell cycle and the egg-to-embryo transition may influence trafficking dynamics. This is particularly evident in the comparison between Miro1 KO GV-stage oocytes and zygotes, in which oocytes demonstrate a cortical and perinuclear localization while in zygotes the mitochondria aggregate in a large perinuclear mass. This indicates a switch to dynein-mediated trafficking after fertilization and possibly a reduced role for short-range actin-mediated dispersion/anchoring of mitochondria through the cytoplasm. These differences in oocytes and zygotes no doubt reflect a complex interplay of motor proteins with multiple mitochondrial adaptors and the cytoskeleton across multiple cell cycle states (Chung et al., 2016; Moore and Holzbaur, 2018).

Interestingly, we have identified that MIRO1 is required for the maintenance of normal mitochondrial size and morphology in oocytes. The enlarged size and complicated cristae structures in *Miro1* deleted oocytes are consistent with several studies in different systems indicating that MIRO1 protein regulates mitochondrial structure. Yeast cells lacking Gem1p (yeast MIRO) contain enlarged, collapsed, globular, or grape-like mitochondria (Frederick et al., 2004), while in HeLa cells, MIRO1 senses cytosolic Ca^2+^ and undergoes a mitochondrial shape transition (MiST) from spaghetti-like to doughnut-like independent of mitochondrial fission machinery (Nemani et al., 2018). The roles of these MIRO1-mediated changes in structure are unclear but appear to provide a means of responding to the physiological state of the cell and cell signaling pathways.

The functional consequences of these changes in the mitochondrial organization have been examined *in vitro* and *in vivo*. Oocyte maturation *in vitro* is compromised with Miro1 KO oocytes showing no effect on GVBD but a 20% reduction in the first polar body extrusion. Persistent levels of Cyclin B1-GFP suggest this is largely due to an increase in arrest at the metaphase of meiosis I. This increase in the rate of arrest is associated with changes in mitochondrial distribution during maturation but a causal relationship needs further testing. Other models that disrupt mitochondrial localization in oocytes include *Mfn1/2* knockout and overexpression, both of which lead to aggregated mitochondria and disrupted meiotic maturation. The effects of the *Mfn1/2* conditional knockouts may lie more in the disruption of mitochondrial function than localization (Wang et al., 2020), while MFN1 overexpression, like loss of MIRO1, led to increased mitochondrial aggregation and an increased arrest at metaphase I with disrupted spindles (Wakai et al., 2014). *Miro1*-deleted oocytes in contrast showed relatively normal spindles suggesting that mitochondrial aggregation itself does not adversely affect spindle formation or oocyte maturation and that *Mfn1/2* overexpression may lead to other mitochondrial defects that cause arrest at the MI stage.

After fertilization, most zygotes show a dispersed mitochondrial distribution with some level of perinuclear accumulation (Nagai et al., 2004; Dumollard et al., 2007). This is markedly exacerbated in *Miro1*-deleted zygotes and 2-cell embryos. This change in distribution did not alter the ability to segregate mitochondria equally to daughter blastomeres at the 2-cell stage. To avoid any contribution from the paternal allele we examined the development of diploid parthenotes to the blastocyst stage. There was a 20% reduction in blastocyst formation in the absence of MIRO1 but no specific developmental event appeared to be disrupted and blastocysts that did develop were similar in size to controls. The dramatic decrease in *Miro1* mRNA levels between the oocyte and 2-cell stage may be an indicator that MIRO1 is more prevalent in oocytes than in preimplantation embryo development.

Despite the high level of *Miro1* expression in oocytes, the lack of MIRO1 during oocyte growth and up to the point of embryonic gene expression appeared to have little effect on fertility since similar numbers of pups were generated in control and Miro1 KO mice. Although the biological variation associated with breeding studies means small differences in oocyte fertility may go unnoticed, the decrease in maturation and development *in vitro* indicates the absence of MIRO1 may render oocytes and embryos more sensitive to *in vitro* conditions.

Manipulating mitochondrial adaptor proteins to sever mitochondria from motor proteins may yet prove to be an incisive means of addressing the role of mitochondrial localization on events of oocyte maturation and embryo development. MIRO1 clearly plays a role in the distribution of mitochondria in oocytes and zygotes, but in its absence, other mechanisms for hitching mitochondria to the cytoskeletal network persist. Whether this is compensation by MIRO2 and/or TRAK-mediated coupling to other mitochondrial membrane proteins remains to be determined.

Nevertheless, the fact *in vitro* oocyte maturation and embryo development is compromised in the absence of MIRO1, suggests its expression in oocytes affords some optimization of oocyte function to support maturation and development.

## Data Availability

The datasets presented in this study can be found in online repositories. The names of the repository/repositories and accession number(s) can be found below: GEO database under the accession number GSE97778.
