# Supplementary material for: Miro1 depletion disrupts spatial distribution of mitochondria and leads to oocyte maturation defects
Source: Front Cell Dev Biol. 2022 Oct 17;10:986454. doi: 10.3389/fcell.2022.986454 (PMC9619047; doi:10.3389/fcell.2022.986454)
Supplement: Supplementary file 1 [file Table1.DOCX]

Supplementary Material

# Supplementary Data

## Genomic DNA Extraction and PCR

A pool of 20 GV-stage control or Miro1 KO was transferred to 10 µl lysis buffer (50 mM Tris-HCl (pH 8.0), 1 mM EDTA, 0.5% Tween 20, and 200 µg/ml proteinase K). Each sample was incubated at 55℃ for 2 hours, followed by the inactivation of proteinase K at 95℃ for 10 minutes. DNA was amplified by the reaction of 50 µl consisting of 5 µl DNA, 1x High Fidelity PCR buffer, 2 mM MgSO_4_, 0.2 mM dNTPs, 1 U of Platinum Taq High Fidelity (Invitrogen), and 0.2 μM each of following primers:

P1: 5′-CCCTGTGTCGCTGAGGTTGGAAGCTG-3′,

P2: 5′-GAAATGCCACCAGAATCCAGTGGC-3′,

P3: 5′-GTGGAGGCAGGAGGATCAGGAGTTTAAAGTC-3′.

## FAD^2+^ and NAD(P)H Measurement

Signals of FAD^2+^ and NAD(P)H were detected by autofluorescence. Oxidized flavoproteins (FAD^2+^) autofluorescence was collected with a 505-550 nm bandpass filter after exciting with 488 lasers. FAD^2+^ autofluorescence is limited to mitochondria. The reduced nucleotides NAD(P)H were excited by UV light (405 nm) and emission was collected using a 435-485 nm bandpass filter. NAD(P)H autofluorescence is detected in both mitochondria and cytoplasm. The intensity of each signal in images was measured and normalized using Fiji software.

## Reactive Oxygen Species (ROS) Measurement

To measure intracellular ROS, fully grown GV-stage oocytes were incubated in drops of M2 medium containing 20 µM DCFDA/H_2_DCFDA (Cellular ROS assay kit, ab113851, Abcam) at 37℃ for 30 min. After washing three times with a fresh M2 medium, oocytes were placed in the metal imaging chamber. Images were acquired on a Leica SP8 microscope using a 40x water immersion 1.2 NA objective with line accuracy set to 2. The intensity of each signal in images was measured and normalized using Fiji software.

## ATP Measurement

Fully grown GV oocytes (5 pooled/set) were collected and stored in 50 µl filtered ultrapure water and stored at −80 °C until use. To prepare standards, 10^−7^ M ATP standard stocks were obtained from ENLITEN^®^ ATP Assay System Bioluminescence Detection Kit (Promega) and diluted with filtered ultrapure water. ATP levels were analyzed using the Adenosine 5-triphosphate (ATP) Bioluminescent Somatic Cell Assay kit (FLASC, Sigma-Aldrich) according to the manufacturer’s instructions. 100 µl ATP Assay Mix Working Solution was added to a 96-well plate (reaction vial) (M0187, Greiner) and left at room temperature for 3 min. Somatic Cell ATP Releasing Reagent (100 µl), 50 μl filtered ultrapure water and 50 µl sample were added to a new tube and 100 µl transferred to the reaction vial. ATP concentration was measured immediately using a luminometer (BMG, Clariostar, 76G58). Data were normalized to the mean ATP levels of the control group.

## Mitochondrial Membrane Potential (MMP) Measurement

MMP ratiometric analysis was performed using FIJI as previously described {Al-Zubaidi, 2019 #1595}. Oocytes were stained with Mitotracker Green FM (M46760, Invitrogen) and TMRM, and each channel was simultaneously scanned. To set the background signal as not a number, the obtained images were converted into 32-bit and the threshold was adjusted. The TMRM image was divided by the Mitotracker Green image pixel by pixel using the Image Calculation function.

## siRNA mediated Miro2 Knock-down

Miro2-targeting (siMiro2, L-064101-01-0005) and non-targeting (siControl, D-001810-01-05) small interfering RNAs were purchased from Dharmacon^TM^. After microinjection, the oocytes were cultured for 18 hours in M16 medium containing IBMX. They were then transferred to a fresh culture medium for further maturation process in the incubator.

# Supplementary Figures and Tables

## Supplementary Figures


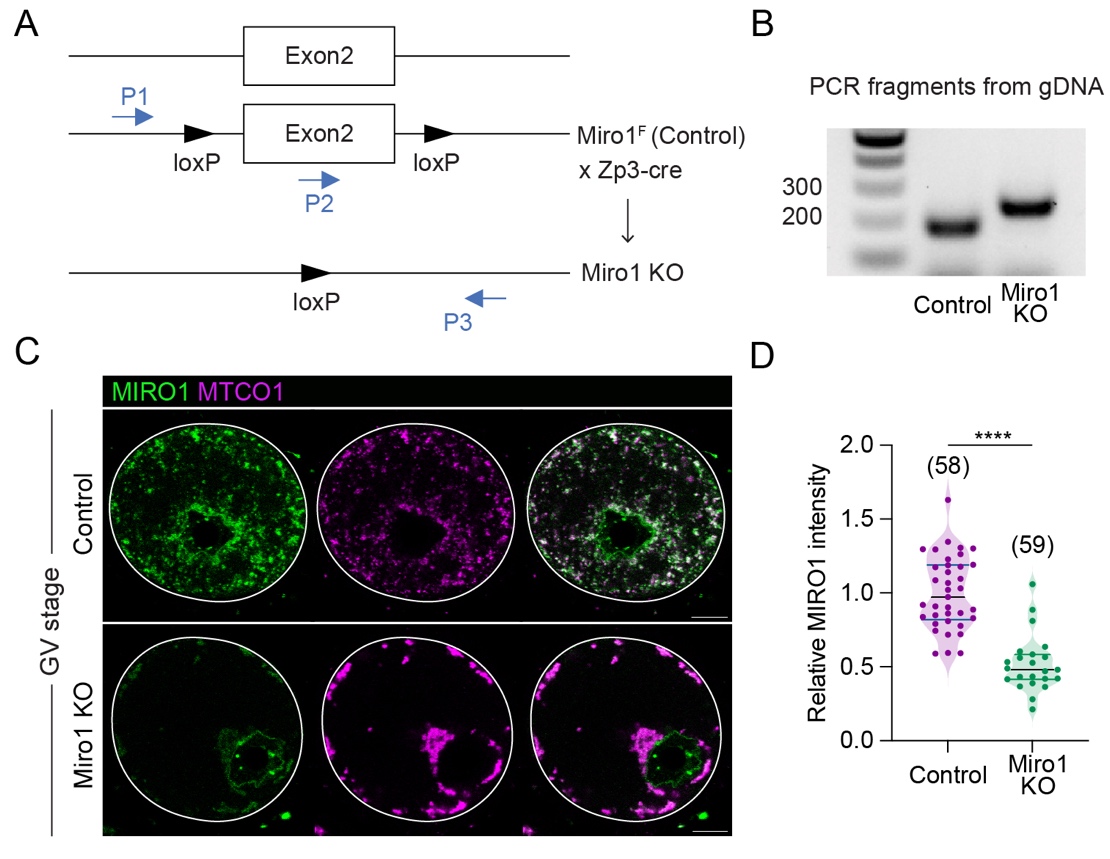


**Supplementary Figure 1**. Generation of *Miro1* null oocytes. **A.** Schematic representation of *Miro1* alleles and deletion of exon 2 and creation of a *Miro1* Δexon 2 allele by ZP3 Cre-mediated recombination in oocytes. P1, P2, and P3 indicate primers for genotyping. **B.** PCR fragments amplified from genomic DNA of GV-stage control and Miro1 KO. **C.** Immunostaining of MIRO1 proteins (Green) and MTCO1 (Magenta) in control and Miro1 KO. **D.** Relative level of MIRO1 signal located at the mitochondria reduced in Miro1 KO compared to that in control. Unpaired t-test ****p<0.0001. Data are collated from 3 independent experimental replicates.


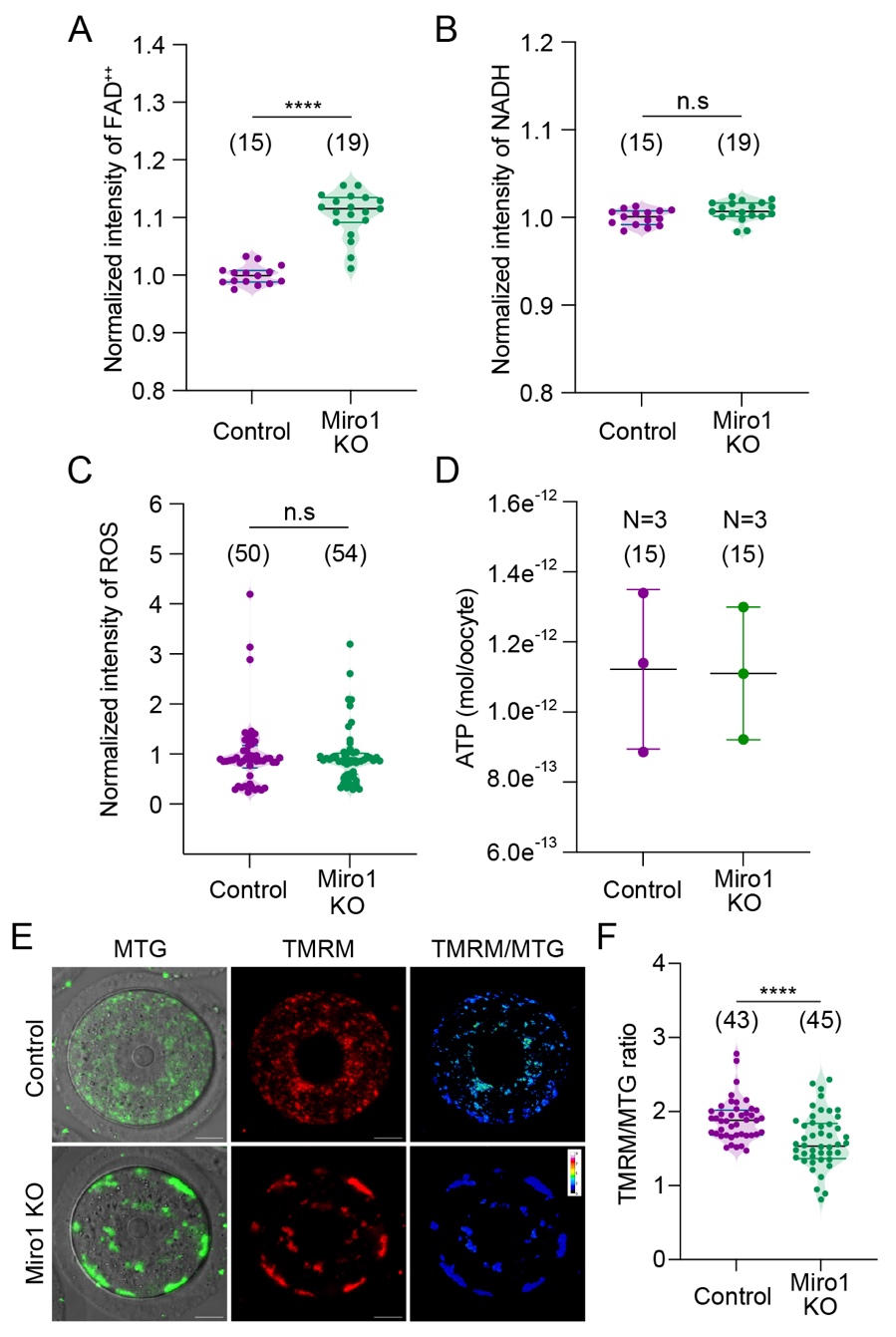


**Supplementary Figure 2.** **Oocyte-specific deletion of *Miro1* marginally disrupts mitochondrial function in oocytes.** **A, B** Normalized levels of FAD^2+^ and NADH contents in control and Miro1 KO. Unpaired t-test ****p<0.0001 and n.s.; not significant. **C.** Normalized level of DCFH fluorescence in control and Miro1 KO. n.s.; not significant. **D.** ATP contents in control and Miro1 KO oocytes. Unpaired t-test n.s.; not significant. Data are collated from 3 independent experimental replicates. **E.** Representative images of the mitotracker green (MTG, green), TMRM (red) and ratiometric mitochondrial membrane potential (blue) in control and Miro1 KO oocytes. Scale bar: 15 µm **F.** Measurement of TMRM/MTG ratio across the cytoplasm in control or Miro1 KO oocytes. Data are collated from 3 independent experimental replicates. Unpaired t-test ****p<0.0001.


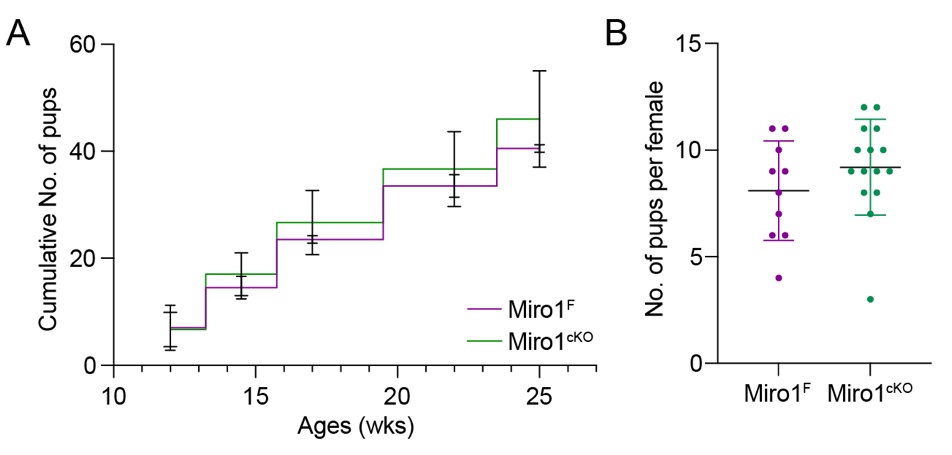


**Supplementary Figure 3.** ***Miro1* conditional knockout mice are fertile.** **A.** Comparison of the cumulative number of pups per *Miro1^F^* (n = 2, purple line) and *Miro1*^cKO^ (n = 3, green line). **B.** Average number of pups per female during the testing period. Results show mean ± SD.


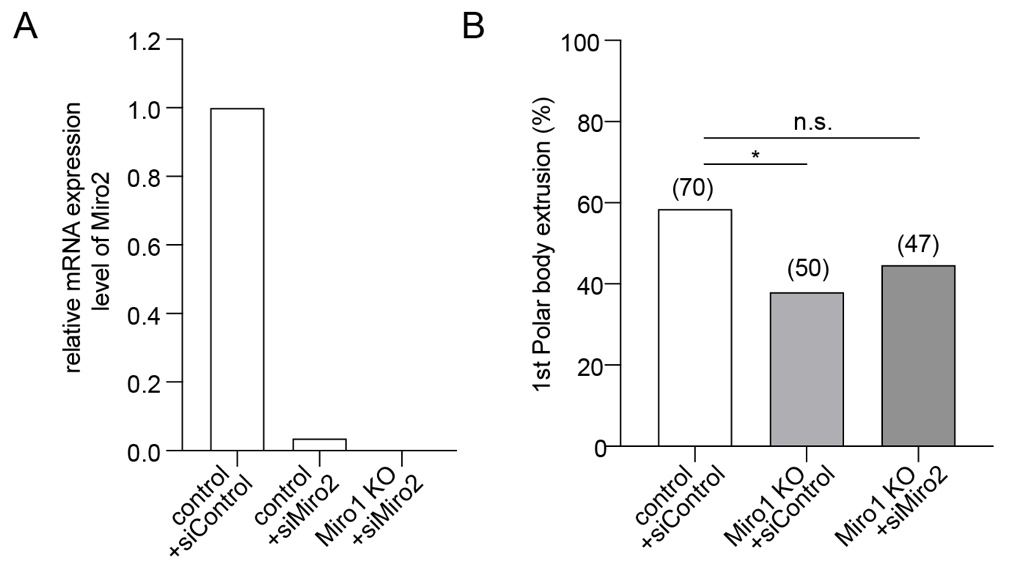


**Supplementary Figure 4. Miro1/2 depletion has no impact on *in vitro* oocyte maturation.** (A) Relative mRNA expression level of Miro2 in control and Miro1 KO oocytes. 30 GV oocytes in each group were microinjected with siControl or siMiro2 and pooled for mRNA isolation. (B) Percentage of the first polar body extrusion in control, Miro1 KO and Miro1/2 depleted oocytes. Chi-square *p<0.05 and n.s., not significant. Data are collated from 3 independent experimental replicates.
